# Reduced heart rate variability and expressive suppression interact to prospectively predict COVID-19 pandemic-related post-traumatic stress symptoms

**DOI:** 10.1038/s41598-022-25915-7

**Published:** 2022-12-09

**Authors:** Carola Dell’Acqua, Francesca Mura, Simone Messerotti Benvenuti, Elisabetta Patron, Daniela Palomba

**Affiliations:** 1 Department of General Psychology, University of Padua, Via Venezia, 8, 35131 Padua, Italy; 2 Padova Neuroscience Center (PNC), University of Padua, Padua, Italy; 3 Hospital Psychology Unit, Padua University Hospital, Padua, Italy

**Keywords:** Psychology, Risk factors

## Abstract

The COVID-19 pandemic is a unique period of stress that, in some cases, led to post-traumatic stress symptoms (PTSSs). Emotion regulation strategies are known to modulate the emotional response to stressful events. Expressive suppression (ES) is a maladaptive strategy related to the exacerbation of the physiological stress response. Heart rate variability (HRV), an index of cardiac autonomic balance strictly related to ES, was also shown to predict PTSSs. This was the first study to investigate whether the pre-pandemic ES use and resting-state HRV predicted pandemic-related PTSSs. Before the pandemic, 83 (58 females) university students completed the Emotion Regulation Questionnaire (ERQ), self-report measures of anxiety and depressive symptoms, and a three-minute resting-state electrocardiogram recording. After 12 months, 61 (45 females) participants completed a self-report measure of pandemic-related PTSSs and repeated the self-report psychological measures. Pre-pandemic anxiety symptoms prospectively predicted greater PTSSs. Moreover, a significant interaction between HRV and ES in predicting PTSSs emerged, whereby those who had higher levels of ES and reduced HRV showed higher PTSSs. These findings suggest that an integrated assessment of HRV and ES might be useful for identifying individuals who are more vulnerable to the development of PTSSs during crises.

## Introduction

Since its outbreak in late 2019, the SARS-CoV-2 (COVID-19) spread worldwide and reached pandemic proportions in March 2020^1^. Multiple factors negatively impacted the mental health of the general population, such as the uncertainty and the fear of contagion, isolation, high mass-media coverage, and the social and economic crises^2^. Several global community-based studies documented elevated levels of anxiety, depression, and post-traumatic stress symptoms (PTSSs) related to the pandemic^1–8^. These symptoms might persist^5^ and develop into full-blown mental disorders. Meta-analytic evidence reported high rates of anxiety (31.9%), depression (33.7%), and stress (29.6%) in community samples globally^9^. The high psychological impact was also thoroughly documented in college students^10–12^, who experience a sensitive period for the development of mental disorders^13^. Because the distress caused by the COVID-19 pandemic, in some cases, led to the onset of post-traumatic stress disorder (PTSD), this global crisis can be considered a specific mass traumatic event^14–17^.

However, in line with the diathesis-stress model^18^, not all individuals that experience traumatic events show consequent psychological distress, but some individuals are more vulnerable to adverse psychological consequences in response to traumatic and stressful events^19^. Following a traumatic event, most individuals develop a temporary stress response, but relatively few individuals experience persistent psychological symptoms^20^. Several factors that predispose individuals to experience elevated and persistent levels of psychological distress when exposed to traumatic events have been identified, such as a family history of psychopathology, poor social support, maladaptive emotion regulation strategies, and prior depressive and anxiety symptoms^21–27^. Although a wealth of evidence suggests that the pandemic is having a negative impact on the population’s psychological well-being, the literature on the psychological factors that could play roles in buffering pandemic-related psychological distress is still lacking.

The way individuals regulate their emotions when provoked by stressful and traumatic events, such as the pandemic, can determine subsequent adverse psychological outcomes. Particularly, emotion regulation strategies are known to modulate the emotional response to stressful events^28,29^. Reappraisal is a cognitive strategy that changes the trajectory of emotional responses by reformulating an event’s meaning^29–31^. This strategy is effective in regulating the emotional stress response. For instance, individuals who habitually use reappraisal are characterized by lower levels of negative affect, reduced experience of negative emotions^31^, and increased resilience^32^. Expressive suppression is a response-focused strategy that consists of inhibiting emotions once they have already been generated^31^. Following traumatic events, the use of expressive suppression might prevent the necessary emotional processing of the event, leading to the development and persistence of symptoms^33,34^. In addition, a few longitudinal studies have demonstrated that expressive suppression predicts PTSS development and reduced psychosocial functioning after stressful and traumatic events^35–37^. Other studies reported that the habitual use of expressive suppression was associated with pandemic-related post-traumatic symptoms^38–41^. Notably, this maladaptive strategy does not lead to a reduced emotional experience; instead, it impedes the expression of emotions, whilst physiological response can remain elevated. It has been shown that the use of expressive suppression can be associated with somatic arousal and physiological strain^42,43^.

A peculiar autonomic measure inversely related to impaired emotion regulation and associated positively with efficient emotion regulation is heart rate variability (HRV^44–50^). HRV is defined as the fluctuations in interbeat intervals over time, and it is considered a reliable measure of cardiac autonomic balance and reflects overall physical health^51^. Low resting HRV was shown to be associated with emotion dysregulation^52^ and, more specifically, with higher use of maladaptive emotion regulation strategies^44,53–56^. An explanation for this association could lay in the evidence that HRV is believed to be modulated by a network that involves brain structures known for emotion regulation (i.e., central autonomic network, CAN^49,57–60^). Notably, individuals with PTSD have greater cardiovascular risk^61,62^ and a reduced HRV^63,64^. Additionally, longitudinal studies demonstrated that cardiovascular diseases^65^ and low HRV before a traumatic event predicted greater PTSSs in response to the event^66–69^. Individuals with lower HRV during the pandemic showed significant reductions in psychological well-being, compared to those with higher HRV^70^. In addition, a longitudinal study on a sample of adolescents reported that the link between psychological distress and pandemic-related affective symptoms was moderated by HRV assessed before the pandemic^71^. However, a limited number of longitudinal studies were conducted to assess the predictive role of reduced HRV in the development of psychological distress due to a traumatic event^67–69^. Even though HRV appears to be related to the stress response, the nature and direction of this association are still unclear.

Given the individual differences in response to trauma-related stressors, the lack of longitudinal studies examining the interaction of psychological and autonomic measures in predicting maladaptive stress responses is surprising. Indeed, as indicated by the above-reviewed literature, emotion regulation and HRV are both associated with the response to stressors (e.g.^35,42,44,67–69,71^). The investigation of premorbid features is crucial to identify individuals who might suffer from severe consequences following trauma exposure and who might benefit from close monitoring and treatment following exposure to a stressor. To address this gap, this longitudinal study’s objective was to investigate the role of pre-pandemic expressive suppression, reduced HRV, and the interaction of these in predicting pandemic-related PTSSs. The present study examined a sample of undergraduate college students, as they are considered a particularly vulnerable population to the development of distress following stressful life events^10–13^. A moderating role of HRV in the association between expressive suppression and pandemic-related PTSSs was expected, whereby those with lower HRV and greater habitual use of expressive suppression would show greater pandemic-related PTSSs. The role of pre-pandemic cognitive reappraisal, anxiety, and depressive symptoms was taken into account in the analyses.

## Methods

### Participants

The present study was conducted within an extensive research project, and some participants’ data was already presented in previous publications (see^72–74^). Eighty-three (58 females) Italian Caucasian students at the University of Padua voluntarily took part in the study. A convenience sampling method was used: the sample was recruited from several large introductory courses in psychology at the University of Padua, Italy. Exclusion criteria included a past or current history of cardiovascular, psychiatric, and neurological diseases, as well as the intake of psychotropic medications. All participants were right-handed and had normal or corrected-to-normal vision. Participants were naive to the aim of the study and received monetary compensation for their participation. Of the original 83 participants who took part in the pre-pandemic assessment (first assessment, December 2019–February 2020), 61 (45 females) took part in the follow-up, which occurred 12 months after the first assessment (December 2020–February 2021). The attrition rate of this longitudinal study was within the expected range (26.5%)^75^, and participants’ reasons for dropouts were not registered. The present study was conducted with participants’ adequate understanding and written consent in accordance with the Declaration of Helsinki and was approved by the University of Padua’s local ethics committee (prot. no. 3612).

### Procedure

The present study involved two experimental sessions. The first assessment took place in the psychophysiological laboratory of the Department of General Psychology at the University of Padova between December 2019 and February 2020. After giving written informed consent, participants were administered an ad hoc anamnestic interview and three psychological questionnaires (Beck Depression Inventory-II, BDI-II; Beck Anxiety Inventory, BAI; Emotion Regulation Questionnaire, ERQ). Subsequently, participants were seated on a comfortable chair in a dimly lit, sound-attenuated room, and a three-minute resting electrocardiographic was recorded. The second experimental session was conducted online, approximately a year after the first visit, between December 2020 and February 2021. The second assessment included the administration of the same three psychological questionnaires and the Impact of Event Scale (IES-R), a commonly used tool to measure PTSSs related to any traumatic event^76^.

## Measures

### Psychological assessment

Habitual use of expressive suppression and cognitive reappraisal was collected at both assessments using the ERQ^77,78^. The ERQ is a self-report questionnaire to assess the respondent’s tendency to use cognitive reappraisal or expressive suppression as a strategy to modulate one’s emotional responses. Cognitive reappraisal is an emotion regulation strategy that consists of re-evaluating an emotion-eliciting situation to change the meaning of the event and reduce its impact. On the other hand, expressive suppression consists of reducing the experiential and behavioral aspects of emotion. The measure is composed of 10 items that refer to the use of cognitive reappraisal (e.g., “I control my emotions by changing the way I think about the situation I’m in”) or expressive suppression (“I control my emotions by not expressing them”). Each item is rated on a 7-point Likert scale from “strongly disagree” to “strongly agree.” The questionnaire provides two scores, one for cognitive reappraisal (ERQ-R) and one for expressive suppression (ERQ-S). Higher scores indicate greater employment of the emotion regulation strategy^77,78^.

To assess post-traumatic symptoms due to the COVID-19 pandemic, the IES-R^76,79^ was used exclusively during the follow-up assessment. The IES-R is a self-report measure composed of 22 items that refer to post-traumatic symptoms (e.g., insomnia and intrusive thoughts) provoked by any traumatic event. In this case, participants were asked to refer to the COVID-19 pandemic (e.g.^80,81^). Respondents are asked to rate how distressing each item has been during the previous week on a Likert scale from 0 (“not at all”) to 4 (“extremely”). Scores lower than or equal to 23 indicate minimal post-traumatic stress symptoms, whereas scores above 24 indicate the presence of clinically significant PTSSs. Particularly, scores greater than 24 can be divided into mild-to-moderate symptomatology (ranging from 24 to 36) and severe symptomatology (higher than 37)^76,79,82^.

The BDI-II^83,84^ was used at both assessments to measure the severity of depressive symptoms. Each of the 21 items is comprised of a group of statements that address a particular depressive symptom (e.g., punishment feelings and loss of interest). Respondents are asked to read each statement and select the one that best describes how they have felt for the past two weeks. The statements are scored on a scale from 0 to 3 depending on their level of severity, with a higher sum (range 0–63) suggesting more severe symptoms^84^.

The BAI^85,86^ was used at both assessments to measure anxiety levels. The BAI includes 21 items, each based on a 4-point Likert scale, and scores range from 0 to 63. Whereby, greater scores indicate greater anxiety symptoms^86^.

### Electrocardiographic recording

The electrocardiographic (ECG) signal was acquired using three Ag/AgCl electrodes positioned according to Einthoven’s lead II configuration. ECG recordings were collected at rest for 3 min, sampled at 1000 Hz, and band-pass filtered (0.3–100 Hz). The ECG signal was visually inspected and corrected for artifacts using a piecewise cubic spline interpolation method to generate missing or corrupted values into the normal-to-normal (NN) intervals. Then, R-peak detection was used to compute interbeat intervals and mean heart rate (HR). HRV indices were computed using Kubios HRV Analysis software 2.2 (MATLAB, Kuopio, Finland). Specifically, time-domain HRV indices were calculated through autoregressive spectral analysis. The standard deviation of the normal (NN) sinus-initiated interbeat intervals (SDNN) in ms was computed because it is considered a reliable HRV index in short-term recordings^87^. To fit the assumptions for linear analyses, the SDNN was natural log-transformed^88^. The SDNN is a widely used and accurate measure of total HRV, especially in short-term recordings. In resting conditions, its primary source of variation is parasympathetically mediated respiratory sinus arrhythmia^87^.

### Statistical analysis

All analyses were performed using RStudio Version 1.4.1717. As a preliminary analysis to assess potential changes in emotion regulation (ERQ-S, ERQ-R), anxiety (BAI), and depression scores (BDI-II) between the first assessment and the follow-up, two-sided paired samples *t*-tests were conducted. Considering that the male sex was underrepresented, an independent *t*-test was conducted to examine whether sex influenced the dependent variable of interest (IES-R).

Then, Pearson correlations were performed for each study variable collected at baseline (BDI-II, BAI, ERQ-S, and ERQ-R) and IES-R scores. The Benjamini–Hochberg procedure was applied to control the false discovery rate in the correlations^89^.

Finally, a multiple linear regression model was conducted to assess the main effect of pre-pandemic expressive suppression scores, HRV, and the interaction of these in predicting psychological distress due to the pandemic (assessed at the 1-year follow-up). Pre-pandemic BDI-II, BAI, and ERQ-R scores, as well as sex, were included in the model as covariates to avoid confounding effects. All variables were centered and scaled: the mean of each variable was subtracted by each value, and the resulting value was then divided by the standard deviation of its distribution. Using the *mctest* package^90^, multicollinearity diagnostics were run by calculating the variance inflation factors (VIF). A *p*-value of 0.05 was the cut-off for significance. Significant interactions were explored by conducting a simple slope analysis at the mean and 1 standard deviation (SD) below and above the mean using the interactions package^91^.

## Results

### Characteristics of the sample

A sensitivity power analysis in G*Power^92^ for a linear multiple regression model with seven predictors was performed to determine whether the sample size was large enough to detect a significant effect. This analysis revealed that the sample size was large enough to detect a moderate effect size (*d* = 0.56) with a power of 0.80.

Regarding the demographic characteristics, there were no differences in age (*p* = 0.40; initial sample: M_age_ = 20.5, SD_age_ = 2.5; follow-up sample: M_age_ 20.6, SD_age_ = 2.7) and gender (*p* = 0.20) between the subsample that participated in the follow-up and the subsample that did not. Regarding the psychophysiological measures, there were no differences in BAI scores (*p* = 0.666), BDI scores (*p* = 0.70), ERQ-S scores (*p* = 0.47), ERQ-R (*p* = 0.10) scores, and HRV (lnSDNN; *p* = 0.46) between the subsample that participated in the follow-up and the subsample that did not.

The IES-R, the average score was 26.0 (SD = 16.9), and the scores ranged from 2 to 88. Particularly, 34 participants had a score below 23 (absence of symptoms); 12 participants had a score between 24 and 36 (mild-moderate PTSSs); and 15 had a score greater or equal to 37 (severe PTSSs). Table 1 illustrates the descriptive statistics of self-report measures at the baseline and follow-up assessments, as well as the changes. There were no significant changes in the scores of the ERQ-S and ERQ-R between the two assessments. Moreover, there was a marginally significant increase in depressive symptoms from the first to the second assessment.

**Table 1:** Self-report measures of depressive symptoms (BDI-II), anxiety symptoms (BAI), expressive suppression (ERQ-S), and reappraisal (ERQ-R) at baseline and at the one-year follow-up.

	Baseline	Follow-up	*p*
BDI-II	10.4 (9.0, 0–41)	11.8 (10.2, 0–45)	0.07
BAI	11.6 (9.2, 0–43)	9.3 (9.3, 0–38)	**0.01**
ERQ-S	13.8 (5.71, 4–27)	14.7 (5.72, 4–26)	0.20
ERQ-R	28.5 (6.22, 11–42)	28.4 (6.18, 14–42)	0.67

Significant changes are in bold.

*BDI-II* Beck Depression Inventory, *BAI* Beck Anxiety Inventory, *ERQ-S* Emotion Regulation Questionnaire-Expressive Suppression scale, *ERQ-R* Emotion Regulation Questionnaire-Reappraisal scale.

Regarding symptoms of anxiety, results showed a significant difference in BAI scores between the two assessments, specifically displaying milder symptoms of anxiety in the follow-up, with respect to the first assessment. Concerning the independent sample *t*-tests examining sex differences in IES-R scores, a significant difference emerged, *t*(59) = 3.03, *p* = 0.004, showing higher PTSSs in females, compared to males. For this reason, sex was included in the regression model as a covariate to avoid confounding effects.

The average HRV values of the analyzed sample were 3.86 (SD = 0.45), which were within normative values^93^.

### Correlations between baseline measures and IES-R

Correlations among baseline variables and IES-R scores are displayed in Table 2. Depressive symptoms (BDI-II), anxiety symptoms (BAI), and expressive suppression (ERQ-S) were positively correlated with PTSS levels (IES-R), whereas HRV (lnSDNN) and reappraisal (ERQ-R) were not correlated with PTSSs.

**Table 2:** Pearson’s correlations among study variables.

	BAI_t0_	BDI_t0_	ERQ-R_t0_	ERQ-S_t0_	lnSDNN_t0_
BAI_t0_	–	–	–	–	–
BDI_t0_	0.74*	–	–	–	–
ERQ-R_t0_	− 0.12	− 0.05	–	–	–
ERQ-S_t0_	0.27*	0.32*	− 0.21	–	–
lnSDNN_t0_	− 0.15	− 0.19	0.03	− 0.02	–
IES-R	0.58*	0.47*	− 0.10	0.30*	− 0.05

*BAI* Beck Anxiety Inventory, *BDI-II* Beck Depression Inventory, *ERQ-R* Emotion Regulation Questionnaire-Reappraisal scale, *ERQ-S* Emotion Regulation Questionnaire-Expressive Suppression scale, *lnSDNN* natural logarithm of standard deviations of N–N intervals. All variables were scaled and centered, and *p*-values were FDR Benjamini–Hochberg Adjusted.

*p < 0.05.

### The role of pre-pandemic expressive suppression and HRV in predicting pandemic-related PTSSs

**Figure 1:**
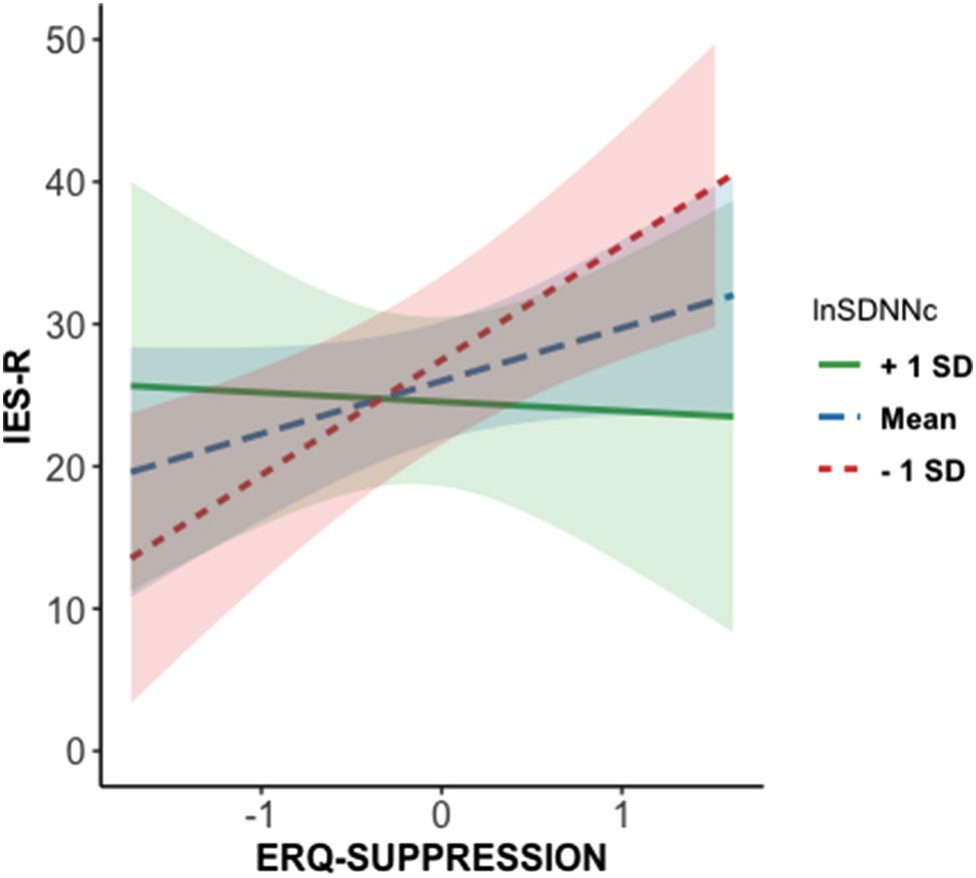
Interaction effect of lnSDNN and ERQ-S on pandemic-relates PTSS (IES-R scores). When lnSDNN is equal to − 1SD, a significant association between ERQ-S and IES-R is displayed (*p* = 0.01). On the other hand, no significant effect emerges when lnSDNN is equal to the mean (*p* = 0.46) or + 1SD (*p* = 0.42). *Note*. + 1 SD, mean and − 1 SD lnSDNN are presented in different colors. Shaded areas represent the standard error of the estimate of the interaction. All variables were scaled and centered.

Considering that cognitive reappraisal did not predict IES-R scores, its interaction with HRV was not included in the model. Results from the regression model are displayed in Table 3. A significant effect of the ERQ-S × HRV interaction term emerged. Table 4 summarizes the results of the simple slope analysis. Specifically, the simple slope analysis showed that the effect of expressive suppression in predicting greater pandemic-related PTSSs was only significant when HRV was 1 SD below the mean (Fig. 1). In contrast, no significant relation between ERQ-S and IES-R was found when lnSDNN was equal to the mean or 1 SD higher than the mean. In addition, a significant effect of BAI was found, whereby higher pre-pandemic anxiety symptoms were related to greater pandemic-related PTSSs at the follow-up. No effect of sex, cognitive reappraisal, or depressive symptoms were found. Multicollinearity diagnostics showed tolerable levels of collinearity between the considered variables (VIF < 4).

**Table 3:** Linear regression model testing the main effects of pre-pandemic expressive suppression, HRV, their interaction, and pre-pandemic reappraisal, anxiety, and depressive symptoms as covariates.

Predictor	R^2^	b	Std. Err	[95% CI]	*p*
	0.40				
Sex		− 0.45	0.25	[− 0.95, 0.05]	0.08
BAI		0.49	3.23	[0.19, 0.80]	0.002*
BDI-II		− 0.03	0.18	[− 0.38, 0.33]	0.87
ERQ-R		− 0.02	0.10	[− 0.22, 0.17]	0.81
ERQ-S		0.10	0.11	[− 0.14, 0.34]	0.41
lnSDNN		0.04	0.12	[− 0.20, 0.27]	0.76
ERQ-S × lnSDNN		− 0.28	0.14	[− 0.56, − 0.01]	0.044*

*BAI* Beck Anxiety Inventory, *BDI-II* Beck Depression Inventory, *ERQ-R* Emotion Regulation Questionnaire-Reappraisal scale, *ERQ-S* Emotion Regulation Questionnaire-Expressive Suppression scale, *lnSDNN* natural logarithm of standard deviations of N–N intervals.

*p < 0.05.

**Table 4:** Results of the simple slopes analysis on the moderating effect of HRV (lnSDNN) in the prediction of PTSS (IES-R) from expressive suppression (ERQ-S) scores.

lnSDNN	*b*	Std. Err	[95% CI]	*t*	*p*
− 0.87 (− 1 SD)	0.34	0.13	[1.39, 10.44]	2.62	0.01*
0.06 (Mean)	0.08	0.12	[− 2.85, 5.67]	0.67	0.51
0.98 (+ 1 SD)	− 0.18	0.21	[− 10.44, 4.26]	− 0.84	0.40

*lnSDNN* natural logarithm of standard deviations of N–N intervals, *SD* standard deviation, *CI* confidence intervals.

*p < 0.05.

## Discussion

This longitudinal study’s objective was to investigate the role of the interaction between pre-pandemic expressive suppression and reduced HRV in predicting pandemic-related PTSSs. The study was based on the evidence that the premorbid habitual use of expressive suppression and a reduced HRV have independently been related to greater PTSSs following a traumatic event (e.g.^39–41,67–69,71^). However, given that HRV and emotion regulation are strictly associated (e.g.^44^), their interaction may buffer a detrimental psychological outcome following elevated stress. The present results showed that greater expressive suppression and reduced HRV interacted to predict greater pandemic-related PTSSs prospectively.

As hypothesized, among individuals who habitually used expressive suppression in their everyday life, only those with a reduced HRV were at greater risk of developing elevated PTSSs. This result adds to the existing literature that separately reported the role of expressive suppression and reduced HRV in predicting PTSSs by suggesting that the link between these two measures might underlie this complex vulnerability. The habitual use of expressive suppression leads to greater negative emotions and the tendency to avoid stimuli related to the stressor, potentially leading to greater consumption of cognitive resources^35^. This attempt to modulate one’s emotions could lead to excessive arousal, which may lead to reduced HRV. This cascade of mechanisms, if prolonged, could trigger a vicious circle for which the use of suppression and reduced HRV become intertwined in determining a stress response. This finding further supports the role of individual differences in cardiac vagal tone, indexed by HRV, in the modulation of emotion regulation^48,49^. Broadly, this result is in line with the diathesis-stress model^18^, as it suggests that psychological distress may result from complex interactions between trait vulnerability factors—psychological and physiological—and stressful conditions.

Notably, expressive suppression and HRV alone did not predict PTSSs. Instead, the maladaptive emotion regulation strategy was a significant predictor of PTSSs only in association with HRV. Indeed, individuals with elevated levels of expressive suppression but with higher HRV were less likely to develop greater PTSSs. This is in line with previous studies that demonstrated the role of higher HRV levels in protecting individuals who habitually used maladaptive emotion regulation strategies from the development of psychological disorders (e.g.^94^). This data is relevant because it underlies the importance of combining self-report psychological measures with psychophysiological measures in the assessment of PTSS risk.

In line with previous studies (e.g.^24,95,96^), premorbid anxiety symptoms predicted pandemic-related PTSSs. Hence, this supports that delivering preventive programs aimed at reducing anxiety in at-risk populations might be important for reducing the impact of exposure to aversive situations^97^. Interestingly, the use of expressive suppression and reappraisal did not change across the two assessments, and this provided further support for the notion that emotion regulation strategies can be considered a vulnerability trait^31^. Instead, anxiety levels were slightly reduced from the pre-pandemic assessment to the follow-up. However, BAI scores were within a nonclinical range, thus making it hard to interpret this finding.

Furthermore, unlike expressive suppression, reappraisal was not significantly correlated with IES-R scores. Despite reappraisal and expressive suppression being assessed through the same scale, it is important to note that these measures are independent of each other, and individuals that habitually use one strategy are not more likely to avoid the other one^31^. Indeed, expressive suppression regulates the generation of emotional responses during the event, but reappraisal occurs later and consists of a reinterpretation of the emotional event. Hence, it could be posited that, in this context, maladaptive emotion regulation strategies when processing a stressful event played a more central vulnerability role.

From a clinical perspective, this study highlights the importance of considering the implementation of top-down and bottom-up interventions in the prevention of PTSS onset.

For instance, implementing novel protocols aimed at intervening simultaneously on autonomic and expressive suppression components. Hence, a combined psychophysiological intervention could be key to preventing the development and exacerbation of PTSSs.

The present findings should be considered in light of some limitations. First, the oversampling of some characteristics (i.e., female sex, young age, homogeneous ethnicity, and enrollment in university) might limit the generalization of our findings to the general population. Second, the reasons for participants to dropout were not registered, and future studies should collect this information. Finally, the use of self-report tools for the clinical assessment, the lack of information about the presence of post-traumatic symptoms before the pandemic, and socioeconomic data might have influenced the results. Although the IES-R is a commonly employed and reliable measure of PTSSs^81,98^, a clinical interview could have been implemented to evaluate these symptoms better. Third, the PTSS assessment was performed during the pandemic’s second wave in Italy—several months after the COVID-19 outbreak—and conducting multiple assessments could have provided valuable insight into the course of symptoms. However, assessing PTSSs one year after the pandemic outbreak was fundamental to identifying individuals who were subjected to a higher risk of developing a prolonged maladaptive stress response because a transient stress response following a traumatic event is rather common^20,99^. Future studies should be conducted to explore whether the present findings can be generalized across multiple cultures. For example, it would be interesting to study this phenomenon in East Asian cultures, where expressive suppression is more prevalent and is less likely to have negative consequences^100,101^.

The present study granted novel evidence on psychophysiological vulnerability factors involved in the development of PTSSs following the COVID-19 pandemic. Understanding the psychophysiological underpinnings of vulnerability to PTSSs is essential to developing effective systematic screening programs for the prevention and treatment of PTSSs. Taken together, our evidence suggests that an integrated assessment of HRV and expressive suppression may constitute a more accurate predictor of PTSSs than the two independent measures alone do.

## Data Availability

The datasets analyzed during the current study are not publicly available due to ethical concerns but are available from the corresponding author on reasonable request.
